# Effect of Maternal CD163 Knockout on PRRSV Replication and Vertical Transmission

**DOI:** 10.3390/ani16182937

**Published:** 2026-09-18

**Authors:** Fumiao Han, Cuizhen Wang, Guohao Liang, Xinxin Luo, Jian Zhang, Zhenfang Wu, Huaqiang Yang

**Affiliations:** 1 State Key Laboratory of Swine and Poultry Breeding Industry, National Engineering Research Center for Breeding Swine Industry, College of Animal Science, South China Agricultural University, Guangzhou 510642, China; 20243139027@stu.scau.edu.cn (F.H.); wangcuizhen@stu.scau.edu.cn (C.W.); zhangjian2025@stu.scau.edu.cn (J.Z.); wzf@scau.edu.cn (Z.W.); 2 Wens Foodstuff Group Co., Ltd., Yunfu 527400, China; leunggodho@163.com (G.L.); 13437845452@163.com (X.L.)

**Keywords:** CD163, gene editing, knockout, PRRSV, transplacental infection, vertical transmission

## Abstract

Porcine reproductive and respiratory syndrome virus (PRRSV) depends on the CD163 receptor to establish infection. Although CD163-knockout (KO) pigs exhibit resistance to PRRSV-related respiratory disease, their reproductive performance following viral challenge has not been adequately assessed. In this study, we assessed the resistance of CD163-KO gilts and sows to PRRSV, focusing on both systemic infection and vertical transmission, by performing two independent challenge experiments: one in nulliparous gilts and another in late-gestation dams, all inoculated with a NADC30-like PRRSV strain. In nulliparous gilts, wild-type (WT) animals developed severe viremia, fever, viral shedding, seroconversion, and high tissue viral loads, whereas CD163-KO gilts remained completely asymptomatic with undetectable viral RNA and no antibody response throughout the 21-day observation period. In WT pregnant dams, PRRSV was transmitted to the vast majority of fetuses. Conversely, all CD163-KO dams remained clinically healthy, and their viable fetuses uniformly tested negative for viral antigen, irrespective of fetal CD163 genotype. These results unequivocally demonstrate that maternal CD163 deficiency completely abrogates both PRRSV replication and transplacental spread, preserving reproductive health and fetal viability, thereby confirming that CD163 is indispensable for all major manifestations of PRRSV infection and highlighting the strong potential of CD163-edited pigs for disease control in the pig industry.

## Simple Summary

Maternal reproductive failure is a critical yet understudied outcome of PRRSV infection. Here, we demonstrate that CD163 knockout (KO) in sows confers complete resistance to vertical transmission, a protection that is independent of fetal CD163 genotype. Notably, fetuses from CD163-KO dams remained entirely uninfected at birth, even when heterozygous for the receptor, revealing that blocking maternal permissiveness alone suffices to eliminate the congenital infection component of reproductive failure. By completely abrogating both horizontal and vertical viral transmissions, our findings constitute a significant leap forward from reactive biosecurity measures to proactive genetic-based disease prevention, providing a durable, single-intervention strategy for sustainable pig production.

## 1. Introduction

CD163 is a type I transmembrane glycoprotein belonging to the scavenger receptor cysteine-rich (SRCR) superfamily. The CD163 gene contains 17 exons, with individual SRCR domains largely encoded by separate exons. The SRCR5 domain, encoded by CD163 exon 7, is critical for PRRSV recognition and entry and therefore plays a pivotal role in PRRSV infection of the host [1]. Consequently, engineering the CD163 gene in pigs to confer resistance to PRRSV infection and subsequently breeding such genetically modified pigs represents a promising strategy for combating this economically significant pig disease [2,3,4]. Multiple lines and breeds of CD163-edited pigs have been established by research teams worldwide, with commercial use already approved in countries like the United States and Canada, highlighting the considerable economic potential of this genetic advancement for the pig industry [5,6,7]. Our team was the first in China to report the successful generation of CD163-knockout (KO) pigs. This was achieved via a targeted disruption of CD163 expression through the introduction of insertion/deletion (indel) mutations in exon 7 [4]. Founder pigs harboring the identical genotype were selected to constitute the disease-resistant breeding populations. CD163-KO pigs have been shown to be resistant to PRRSV infection in multiple laboratories, but most of these animal challenge experiments were conducted based on weaned piglets at about 1 month old. However, PRRS is a complex multisystemic disease that, as its name suggests, affects both the respiratory and reproductive systems [8,9]. However, most previous challenge experiments have primarily focused on respiratory symptoms in piglets, while detailed evaluation of reproductive performance in sows has been largely neglected. Therefore, assessing the reproductive performance of CD163-KO pigs in the context of PRRSV infection constitutes another critical criterion for assessing their disease resistance and overall productive traits.

The present study was designed to evaluate the resistance of CD163-KO pigs to PRRSV with a particular focus on reproductive performance. To this end, we performed two independent animal challenge experiments. In the first experiment, nulliparous gilts were inoculated with PRRSV to compare viral infectivity between wild-type (WT) and CD163-KO animals. In the second experiment, late-gestation sows were challenged with the same virus to determine the efficiency of vertical transmission from WT versus CD163-KO dams to their fetuses. Together, these two experiments yielded complementary insights into both horizontal infection and vertical transmissibility, thereby elucidating the role of CD163 in PRRSV-associated reproductive failure. This study complements earlier research on CD163 editing against PRRS-associated symptoms in weaned piglets and provides evidence for the protection of sow reproductive traits, thereby enabling a comprehensive evaluation of the antiviral function of this gene-edited pig line.

## 2. Materials and Methods

### 2.1. Pigs

CD163-KO pigs were generated via CRISPR-mediated gene editing in pig fetal fibroblasts (PFFs) derived from multiple 30-day-old Yorkshire embryos, followed by somatic cell nuclear transfer (SCNT) using the edited PFFs as donor cells, as described in our previous study [4]. We designed a single gRNA (sequence is GGTCGTGTTGAAGTACAACA with a TGG PAM) for genome cleavage in exon 7 of the pig CD163 gene, which results in frameshift indel in the CD163 exon 7 region. To establish the founder populations, we generated gene-edited pigs via cloning from at least ten unrelated pedigrees to ensure a diverse genetic background. All experimental pigs were raised in Wens Foodstuff Group Co., Ltd. (Yunfu, China). These experiments received approval from the GMO Safety Commission of the Ministry of Agriculture and Rural Affairs, China, and were monitored by the Agricultural and Rural Department of Guangdong Province, China. This study was performed in compliance with the Guiding Opinions on the Treatment of Laboratory Animals (Ministry of Science and Technology of China) and the Constitution of the Institutional Animal Care and Use Committee (IACUC, the Ethics Committee) of South China Agricultural University, and was approved by the Ethics Committee of South China Agricultural University (Approval No. 2025B138).

### 2.2. CD163 Genotyping

CD163-KO pigs were genotyped by amplification of genome sequence around the target site by PCR and then Sanger sequencing of the PCR product. The following primer pair was used for amplifying and sequencing the targeting site of CD163: CD163-F, 5′-GAATTGTCTCCAGGGAAGGA-3′, and CD163-R, 5′-AGCCCAGATCTGTCCACTTC-3′.

### 2.3. PRRSV Infection in Nulliparous Gilts

All experimental infections were performed using a PRRSV NADC30-like strain (GD-7, GenBank accession: OR711915) at a viral titer of 10^7^ TCID_50_/mL [10]. Five CD163-KO and 8 WT nulliparous gilts at the age of 9 months were used for PRRSV infection. Prior to challenge, all animals tested negative for antibodies against PRRSV and for PRRSV antigens, as well as for antigens of other major pathogens, including porcine epidemic diarrhea virus, porcine parvovirus, pseudorabies virus, African swine fever virus, Actinobacillus pleuropneumoniae, and mycoplasma hyopneumoniae. All animals were confirmed to be in good mental and physical condition with normal appetite. On the day of inoculation, each gilt was restrained using a snare rope, and 2 mL of the viral stock was administered intramuscularly into the posterolateral auricular triangle using a 10 mL sterile disposable syringe, with the needle inserted to a depth of approximately 3–4 cm. Following challenge, the gilts were monitored for 21 days; rectal temperatures were recorded at the same time each morning, and clinical signs including lethargy, reduced appetite, dyspnea, and cyanosis of the ear tips and snout were observed and recorded daily. Blood samples from the anterior vena cava and nasal swabs were collected at 0, 3, 5, 7, 10, 15, and 18 days post-infection (DPI). At the end of the observation period, the gilts were euthanized, and tissue specimens, including serum, lung, spleen, tonsil, uterus, and inguinal, mesenteric, and submandibular lymph nodes, were harvested. These samples were divided into two portions, one snap-frozen in liquid nitrogen and the other fixed in 4% paraformaldehyde, for subsequent viral load quantification and histopathological analysis.

### 2.4. PRRSV Infection in Pregnant Sows

For viral challenge in pregnant sows, the pigs used for mating and their genotypes are shown in Table 1. PRRSV inoculation was performed via intranasal instillation on days 90–95 of gestation. Late gestation (gestation day 90 to farrowing) is associated with the highest risk of maternal–fetal transmission and represents the main period during which PRRSV causes reproductive failure [11,12]. Before challenge, each sow was restrained in a farrowing crate with its head fixed. Using a sterile disposable syringe without a needle, 2 mL of the virus stock was slowly instilled into the nostrils (1 mL per nostril), and the head was kept elevated for approximately 30 s after instillation to ensure adequate viral absorption. Between 15 and 19 DPI, all dams were euthanized, and the uterine horns were immediately excised. Fetuses and corresponding placentas were sequentially removed from the tip of each horn and examined for gross pathological abnormalities. The fetal pathological status was scored as follows: 1 = normal; 2 = viable with placental lesions or meconium staining; and 3 = dead and necrotic [13,14]. Sow serum, fetal serum, and placental tissues were collected for virological and histological analyses, while fetal tail tissues were collected for genotyping.

### 2.5. Virological Analysis

Viral RNA was extracted from equal volumes of serum and swab eluates, and from equal weights of tissue, using the HiPure Viral RNA/DNA Kit (Magen, Guanghzou, China). PRRSV RNA levels were quantified via qPCR in accordance with the national diagnostic standard (GB/T 18090-2023) [15]. For absolute quantification of viral RNA copies, a standard plasmid harboring the PRRSV ORF7 gene was constructed and serially diluted to generate the standard curve for the qPCR assays. PRRSV-specific antibody titers in serum samples were determined using the commercial IDEXX PRRS X3 Ab Test kit (IDEXX, Westbrook, ME, USA), with samples exhibiting an S/P ratio of ≥ 0.4 considered positive.

### 2.6. Histological Analysis

For histological analysis, tissue samples were fixed, routinely processed, and embedded in paraffin. Sections were then cut and mounted onto glass slides. For hematoxylin and eosin (H&E) staining, sections were deparaffinized, rehydrated, stained with Harris hematoxylin and eosin, dehydrated, and coverslipped for morphological examination. For immunohistochemistry (IHC), tissue sections were deparaffinized, rehydrated, and then subjected to antigen retrieval by microwave heating in citrate buffer (pH 6.0). Endogenous peroxidase activity was blocked with 3% H2O2. Sections were then incubated overnight at 4 °C with a CD163 primary antibody (clone EDHu-1; GTX42364, GeneTex, Irvine, CA, USA), followed by incubation with an HRP-conjugated mouse IgG Super Vision Assay Kit (Boster, Wuhan, China). Immunoreactivity was visualized using 3,3′-diaminobenzidine (DAB), and sections were counterstained with hematoxylin.

## 3. Results

### 3.1. CD163-KO Gilts Exhibit Complete Resistance to PRRSV Infection

**Figure 1:**
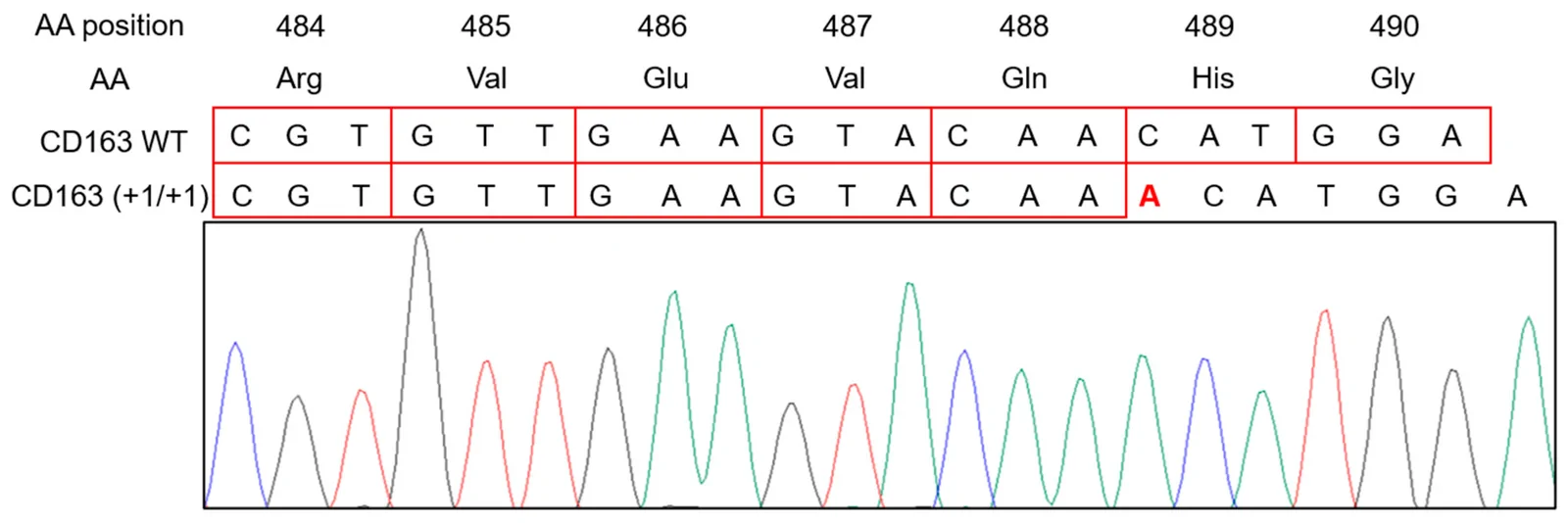
Mutation status of the CD163 gene in CD163-KO pigs. (**Top**) Nucleotide and amino acid (AA) sequence alignment of WT CD163 and the homozygous mutant (+1/+1) spanning amino acid positions 484–490. The red box highlights the WT sequence, while the red A indicates the inserted adenine in the mutant allele, which interrupts normal protein translation and causes a premature stop codon in the following sequence. (**Bottom**) Sanger sequencing chromatogram of the CD163 (+1/+1) mutant. The red A peak corresponds to the inserted nucleotide.

**Figure 2:**
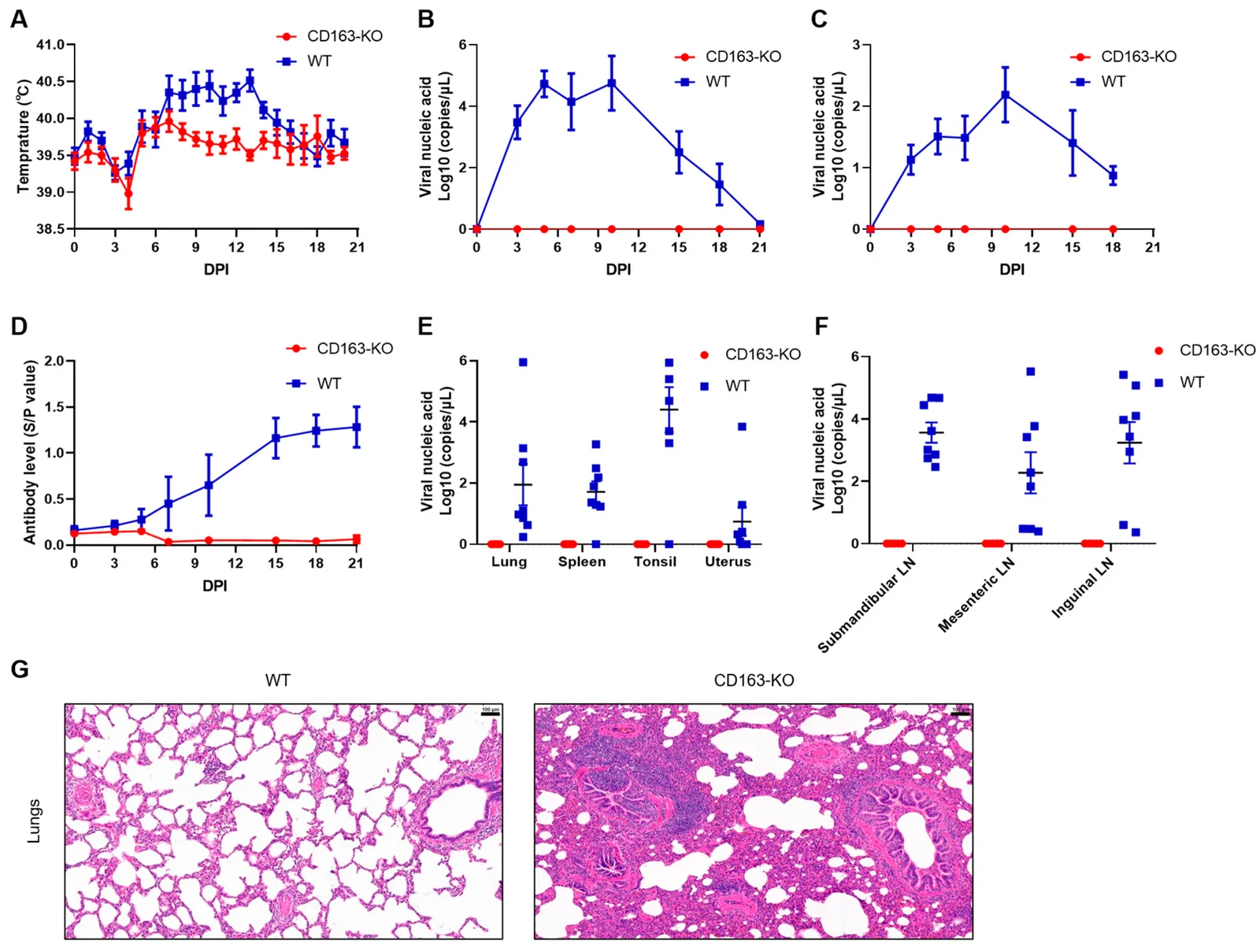
Clinical response, viral load, antibody response and lung pathology in CD163 KO and WT gilts after PRRSV challenge. (**A**) Rectal temperature (°C) over 21 days of infection. (**B**,**C**) Kinetics of viral nucleic acid loads (Log_10_ copies/μL) in serum (**B**) and nasal swabs (**C**) across DPI. (**D**) Serological antibody levels shown as sample to positive (S/P) values over the course of infection. (**E**,**F**) Viral nucleic acid loads in different tissues at study endpoint: (**E**) lung, spleen, tonsil and uterus; (**F**) submandibular lymph node (LN), mesenteric LN, and inguinal LN. Each symbol represents an individual animal; horizontal lines indicate group mean values. (**G**) Representative HE staining of lungs from PRRSV-infected WT and CD163-KO pigs. WT sections exhibit severe perivascular/peribronchial inflammation with vascular cuffs and septal thickening, whereas KO sections have thin septa, open alveoli, and negligible inflammatory changes. Scale bars: 100 µm (**G**).

The CD163-KO gilts used in this study were produced from founder animals that harbor a 1 bp insertion within CD163 exon 7 (Figure 1). As reported in our previous study, these KO pigs exhibit full resistance to PRRSV when challenged in weaned piglets [4]. A total of 13 Yorkshire gilts (5 CD163-KO and 8 WT) at 9 months of age were subjected to viral challenge. All gilts were seronegative for PRRSV antibodies and free of viral antigen at the time of challenge. Following inoculation with PRRSV GD-7 strain, WT gilts developed typical PRRSV-associated clinical signs starting from 3 DPI, characterized by inappetence, lethargy and depression. Fever was first observed in WT gilts at 7 DPI. In contrast, the CD163-KO group remained clinically normal throughout the observation period, with no evident disease signs or fever observed (Figure 2A). One WT gilt died at 13 DPI, while all other gilts survived the full course of the experiment. The gilt died after exhibiting high fever and typical PRRS-associated clinical signs, including cyanosis, lethargy, and anorexia; postmortem examination revealed gross lesions in multiple tissues.

Consistent with the observed clinical disease signs, robust viremia and viral shedding were detected in WT gilts, with viral nucleic acid identified in both serum (Figure 2B) and nasal swabs (Figure 2C). Viral RNA levels increased rapidly following viral challenge, peaked at 5–10 DPI, and subsequently declined gradually. In contrast, viral RNA was undetectable in both serum and nasal secretions from CD163-KO gilts across the entire 21-day observation period. Serum PRRSV-specific antibody responses further differentiated the two experimental groups. WT gilts developed a progressive humoral immune response, with S/P antibody ratios rising continuously from 7 DPI to 21 DPI. By comparison, CD163-KO animals did not elicit any measurable PRRSV-specific antibody response throughout the experiment (Figure 2D). At 21 DPI, viral loads were quantified in a panel of organs and lymph nodes. High levels of PRRSV nucleic acid were detected in the lung, spleen, tonsil, and uterus (Figure 2E), as well as in the submandibular, mesenteric, and inguinal lymph nodes (Figure 2F) of WT gilts. Conversely, virtually no viral RNA was recovered from any of the tissues sampled from CD163-KO gilts. Collectively, these results demonstrate that CD163-KO gilts achieve complete protection against PRRSV challenge, as evidenced by an absence of fever, undetectable viremia, lack of virus-specific seroconversion, and no viral accumulation in susceptible target tissues.

HE staining of lung sections from PRRSV-infected WT pigs revealed marked perivascular and peribronchial inflammatory cell infiltration, with prominent vascular cuffing lesions. Diffuse interstitial inflammation resulted in widespread thickening of the alveolar septa. In contrast, lungs from KO pigs displayed thin alveolar septa, well-aerated alveoli, and only minimal inflammatory infiltrates around bronchi and vessels (Figure 2G).

### 3.2. CD163-KO Dams Protect Fetuses from PRRSV Infection

**Figure 3:**
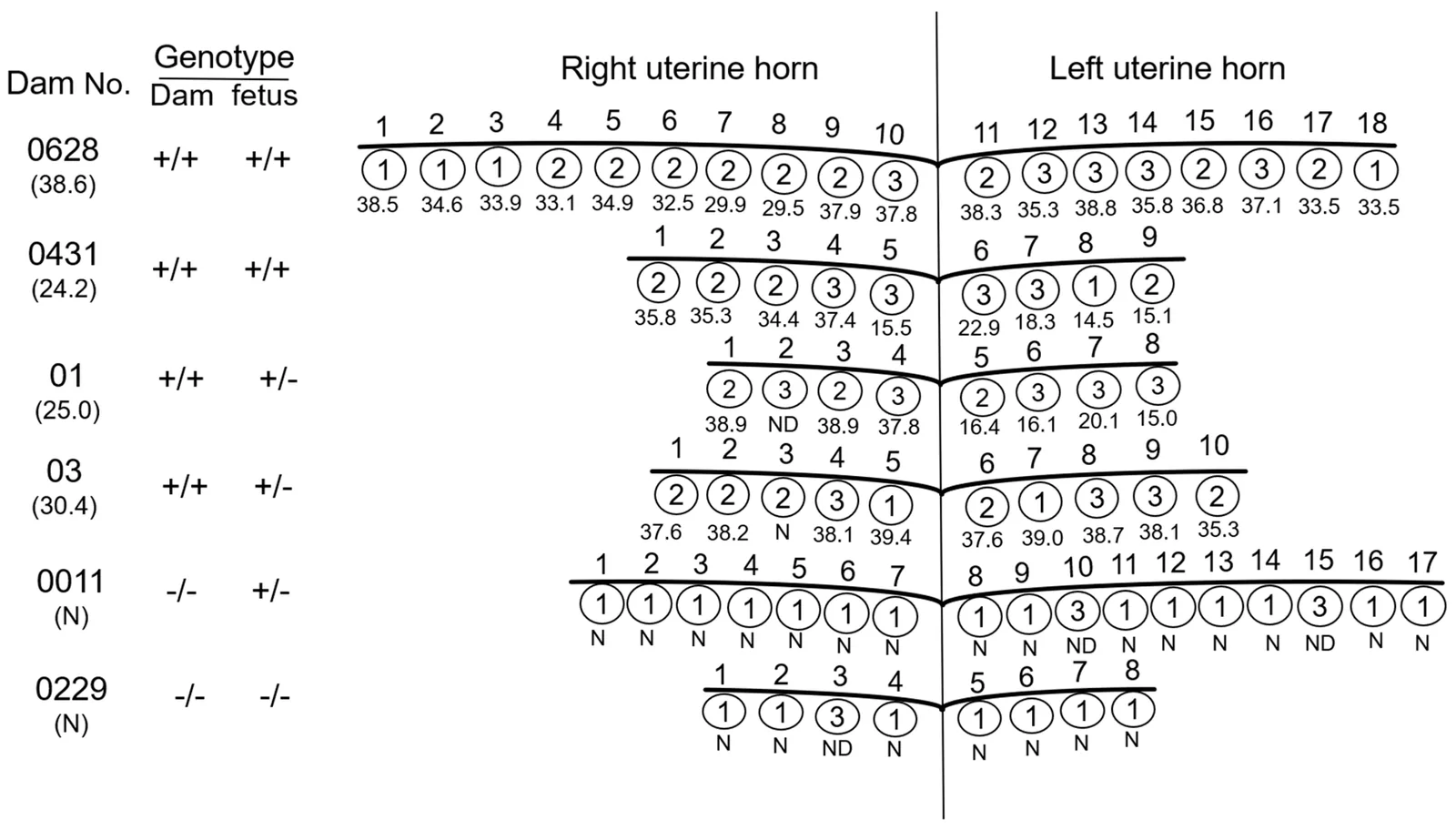
Fetal infection status in PRRSV-challenged sows. Values in parentheses below each sow indicate the PRRSV Ct (antigen level) from maternal serum. Fetuses are labeled by number and position within each uterine horn. Circled numbers below fetuses denote pathological status: 1, normal; 2, viable with placental lesions or meconium staining; 3, dead and necrotic. The lower row of numbers represents PRRSV qPCR Ct values in fetal serum. N, negative for PRRSV RNA; ND, not determined (due to necrosis).

To further evaluate the impact of maternal CD163 deficiency on vertical PRRSV transmission, CD163-KO gilts were mated with either KO or WT boars, while WT gilts mated with KO or WT boars served as controls. The mating pairs are summarized in Table 1. PRRSV infection was performed at gestation days of 90 or 95. Following PRRSV challenge, all WT dams developed clinical signs, characterized by transient lethargy and inappetence, whereas CD163-KO dams remained asymptomatic throughout the observation period. Between 15 and 19 DPI, all dams were euthanized and blood samples were collected from the dams and fetuses (excluding mummified fetuses and amorphous embryos) for subsequent virological analysis. qPCR revealed detectable viremia in all four WT dams, with Ct values ranging from 24.2 to 38.6, while no viral nucleic acid was detected in any of the KO dams. The total number of fetuses recovered per experimental group is detailed in Figure 3 and Table 1. WT dams bred to WT boars (WT ♀ × WT ♂) yielded 18 and 9 fetuses, while WT dams bred to KO boars (WT ♀ × KO ♂) yielded 8 and 10. CD163-KO dams, when mated with WT or KO boars, carried 17 CD163 heterozygous KO (+/-) fetuses and 8 CD163 homozygous KO (-/-) fetuses, respectively. The fetal viremia and gross lesion data are summarized in Figure 3 and Table 2. At the macroscopic level, a substantial proportion (77.8–100%) of fetuses derived from the four WT dams exhibited varying degrees of pathological changes. In the WT ♀ × WT ♂ group, 13 fetuses exhibited placental changes or meconium staining (anatomical pathology grade 2), accounting for 59.1% of all affected fetuses, while 9 fetuses were dead or necrotic (anatomical pathology grade 3), comprising the remaining 40.9%. In the WT ♀ × KO ♂ group, half of the fetuses were classified as grade 2 on anatomical pathology, and the other half as grade 3. These morphological findings are consistent with the typical features of reproductive PRRSV pathogenesis. Notably, the same litters showed a high rate of vertical transmission. Almost all fetuses were positive for PRRSV nucleic acid, with only one negative and one unsampled. In two litters from KO dams, all fetuses appeared grossly normal except for three mummified and malformed fetuses, likely due to developmental issues. Furthermore, all fetuses from KO dams, apart from the three unsampled ones, tested negative for PRRSV antigen (Figure 3, Table 2).

**Figure 4:**
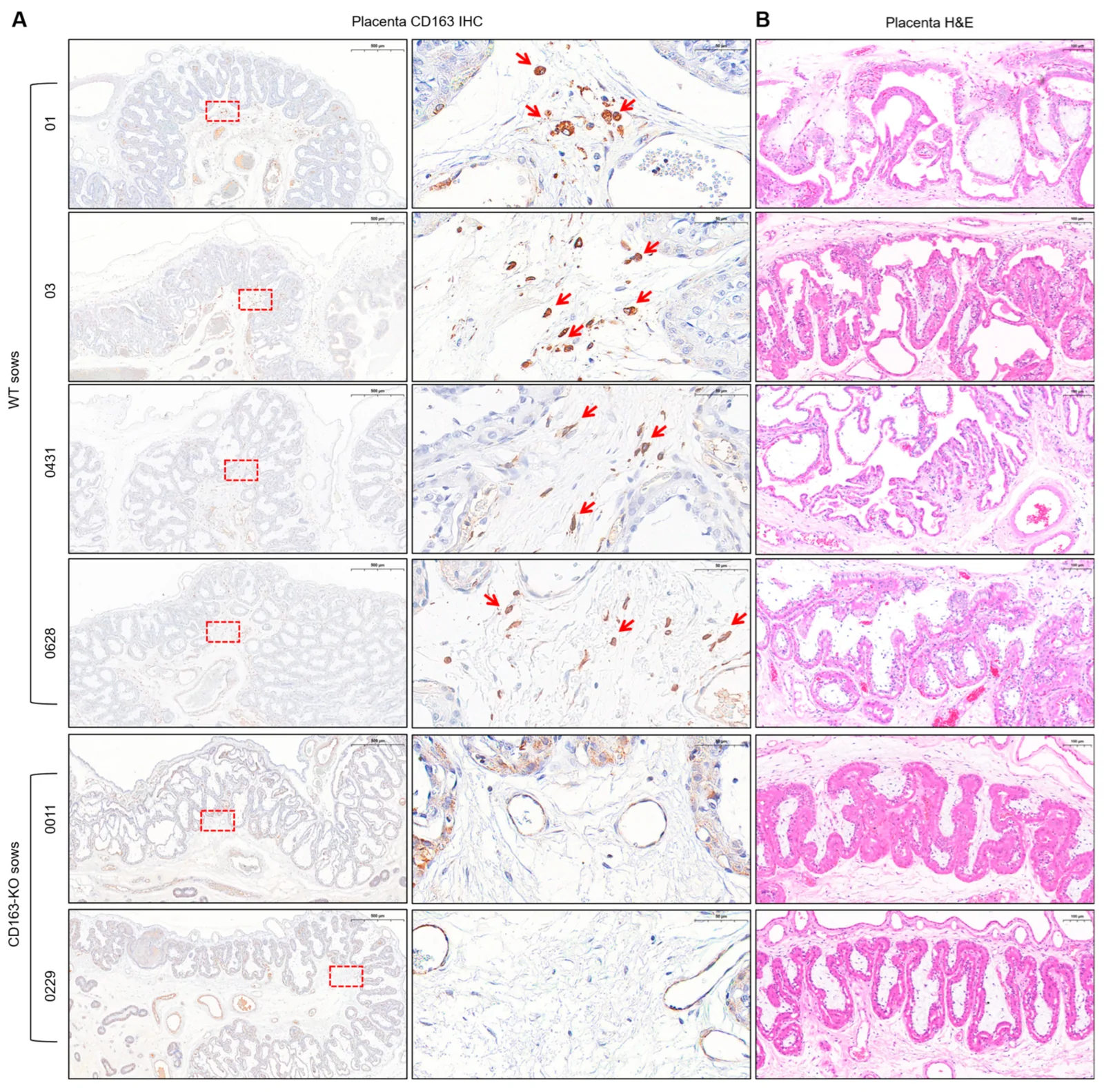
Histological analysis of the placenta. (**A**) IHC staining for CD163 in placental tissues. The right panels show magnified views of the boxed regions in the left panels, highlighting CD163-positive cells (brown staining signals indicated by red arrows). (**B**) H&E staining showing the architecture of chorionic villi. Sows 03 and 01 had the CD163 WT genotype and were mated with CD163-KO males; sows 0431 and 0628 had the CD163 WT genotype and were mated with CD163 WT males; sows 0011 and 0229 had the CD163-KO genotype and were mated with WT and KO males, respectively. Scale bars: 500 µm ((**A**), main), 50 µm ((**A**), inset), and 100 µm (**B**).

We subsequently examined the histological architecture of placentas obtained from all tested dams. IHC staining using a CD163-specific antibody showed scattered CD163-positive cells localized within the decidua basalis of WT sows, suggesting the existence of CD163-positive decidual macrophages that could potentially serve as mediators of PRRSV vertical transmission from the dam to the fetus. In contrast, no such CD163-positive cells were observed in the decidua basalis of placentas from KO sows (Figure 4A). Histological examination by H&E staining demonstrated that the maternal–fetal interface remained structurally intact in the KO group. However, placentas from WT dams displayed pronounced villous detachment and disrupted villous architecture in the intervillous space (Figure 4B).

## 4. Discussion

PRRSV infection causes reproductive failure and respiratory distress. Although pigs of all ages and breeds are susceptible, pregnant sows and piglets are the most vulnerable. The clinical manifestations and pathogenic mechanisms differ markedly between the two groups: sows primarily present with reproductive impairment, whereas piglets predominantly show severe respiratory symptoms [16,17]. In our viral infection experiments using both nulliparous gilts and gestating sows, CD163-KO groups showed a complete absence of PRRSV-associated symptoms, as evidenced by undetectable PRRSV antigen and antibody levels and the blockage of viral transmission to fetuses. In contrast, WT groups, whether mated with WT or CD163-KO males, became infected and transmitted the virus vertically to most fetuses. These findings unequivocally demonstrate that CD163 is essential for PRRSV infection and vertical transmission in vivo. The complete resistance observed in CD163-KO animals confirms that the CD163 receptor is indispensable for PRRSV infection and subsequent pathogenesis in various tissues. Notably, the absence of both antigen and antibody responses indicates that viral replication was entirely abrogated, ruling out subclinical infection. Furthermore, the failure of vertical transmission in CD163-KO dams underscores the importance of maternal permissiveness for transplacental infection. The lack of functional CD163 in the dam prevents viral spread to fetuses, even when the fetuses carry a heterozygous CD163 allele and are thus intrinsically susceptible to PRRSV.

Although the precise mechanisms underlying viral spread across the maternal–fetal interface remain elusive, CD163-positive macrophages are considered critical mediators of PRRSV transmission from the dam to the fetus [18,19]. IHC staining has revealed abundant CD163-positive cells within the placenta, thereby providing a favorable microenvironment for both viral replication and transplacental transmigration during late gestation. The prominent cellular tropism of PRRSV supports the hypothesis that infected macrophages may act as viral “Trojan horses,” facilitating direct cell-to-cell viral dissemination across the placental barrier [9]. Moreover, the virus-induced inflammatory cascade, characterized by the elevated secretion of pro-inflammatory cytokines, is likely to compromise placental integrity by disrupting intercellular tight junctions and promoting apoptosis in adjacent trophoblast cells [20]. Concurrently, the high viral load typically observed in maternal blood during late-gestation viremia may further accelerate barrier breach [9,21]. Collectively, these pathological alterations, ranging from immune cell-mediated shuttling to inflammatory tissue damage, are hypothesized to synergistically overcome the physical and immunological defenses of the placenta, ultimately culminating in vertical transmission and subsequent adverse fetal outcomes, including reproductive failure and late-term abortion.

Besides confirming the critical role of CD163 in PRRSV-associated reproductive syndrome, our viral challenge experiment on gestating sows revealed notable features of fetal PRRSV infection. First, fetuses located in the same uterine region showed similar PRRSV infection levels. For example, in the left uterine horns of dams 0431 and 01, all fetuses had relatively high viral nucleic acid levels, suggesting possible fetus-to-fetus horizontal transmission [22]. Second, viral nucleic acid levels were not directly correlated with fetal pathological severity. For instance, fetus No. 8 from dam 0431 had a PRRSV Ct value of 14.5, but the fetus was generally normal and showed no observable lesions. In contrast, fetus No. 3 from dam 03 was negative for viral nucleic acid but still exhibited grade 2 lesions. These findings indicate that fetal lesions may be primarily attributable to maternal factors rather than to fetal infection itself. Nevertheless, the specific mechanisms remain to be further investigated.

## 5. Conclusions

CD163 KO confers complete resistance to PRRSV infection in gilts and sows, not only eliminating systemic viremia, viral shedding, and viral replication in tissues, but also fully blocking vertical transmission to fetuses during late gestation. These findings conclusively demonstrate that CD163 is the indispensable gatekeeper for both horizontal and transplacental PRRSV spread, and that its genetic ablation can effectively abolish all major pathological manifestations of the disease, including the devastating reproductive syndrome. Our study thus provides strong preclinical evidence supporting the commercial deployment of CD163-edited pigs as a sustainable genetic approach for mitigating PRRSV-associated economic losses.

## Figures and Tables

**Table 1:** CD163 parental and fetal genotypes.

Dam No.	Dam Genotype	Male Genotype	Collection of Fetuses			
			Fetal Genotype	Gestational Age at Infection	Gestational Age at Collection	No of Fetuses
0628	+/+	+/+	+/+	95	110	18
0431	+/+	+/+	+/+	95	110	9
01	+/+	-/-	+/-	90	109	8
03	+/+	-/-	+/-	90	109	10
0011	-/-	+/+	+/-	90	109	17
0229	-/-	-/-	-/-	90	109	8

**Table 2:** Fetal infection status.

Dam No.	Dam Genotype	Male Genotype	Fetus Infection		
			Positive Fetuses	Negative Fetuses	ND ^1^
0628	+/+	+/+	18	0	0
0431	+/+	+/+	9	0	0
01	+/+	-/-	7	0	1
03	+/+	-/-	9	1	0
0011	-/-	+/+	0	15	2
0229	-/-	-/-	0	7	1

^1^ ND, not determined because the fetus was necrotic.

## Data Availability

All data necessary for confirming the conclusions of the article are provided with this paper.
